# Capitalizing on natural language processing (NLP) to automate the evaluation of coach implementation fidelity in guided digital cognitive-behavioral therapy (GdCBT)

**DOI:** 10.1017/S0033291725000340

**Published:** 2025-04-02

**Authors:** Nur Hani Zainal, Regina Eckhardt, Gavin N. Rackoff, Ellen E. Fitzsimmons-Craft, Elsa Rojas-Ashe, Craig Barr Taylor, Burkhardt Funk, Daniel Eisenberg, Denise E. Wilfley, Michelle G. Newman

**Affiliations:** 1 Department of Psychology, National University of Singapore (NUS), Singapore; 2 Technical University of Munich, TUM School of Life Sciences, Freising, Germany; 3 Department of Psychology, The Pennsylvania State University, University Park, PA, USA; 4 Department of Psychiatry, Washington University School of Medicine, St. Louis, MO, USA; 5 Department of Psychiatry and Behavioral Sciences, Stanford University, Stanford, CA, USA; 6 Department of Psychology, Palo Alto University, Palo Alto, CA, USA; 7 Department of Information Systems and Data Science, Leuphana University Lüneburg, Lüneburg, Germany; 8 Fielding School of Public Health, University of California at Los Angeles, Los Angeles, CA, USA

**Keywords:** anxiety, depression, digital mental health intervention, eating disorders, guided internet-delivered cognitive-behavioral therapy, implementation fidelity, machine learning, natural language processing

## Abstract

**Background:**

As the use of guided digitally-delivered cognitive-behavioral therapy (GdCBT) grows, pragmatic analytic tools are needed to evaluate coaches’ implementation fidelity.

**Aims:**

We evaluated how natural language processing (NLP) and machine learning (ML) methods might automate the monitoring of coaches’ implementation fidelity to GdCBT delivered as part of a randomized controlled trial.

**Method:**

Coaches served as guides to 6-month GdCBT with 3,381 assigned users with or at risk for anxiety, depression, or eating disorders. CBT-trained and supervised human coders used a rubric to rate the implementation fidelity of 13,529 coach-to-user messages. NLP methods abstracted data from text-based coach-to-user messages, and 11 ML models predicting coach implementation fidelity were evaluated.

**Results:**

Inter-rater agreement by human coders was excellent (intra-class correlation coefficient = .980–.992). Coaches achieved behavioral targets at the start of the GdCBT and maintained strong fidelity throughout most subsequent messages. Coaches also avoided prohibited actions (e.g. reinforcing users’ avoidance). Sentiment analyses generally indicated a higher frequency of coach-delivered positive than negative sentiment words and predicted coach implementation fidelity with acceptable performance metrics (e.g. area under the receiver operating characteristic curve [AUC] = 74.48%). The final best-performing ML algorithms that included a more comprehensive set of NLP features performed well (e.g. AUC = 76.06%).

**Conclusions:**

NLP and ML tools could help clinical supervisors automate monitoring of coaches’ implementation fidelity to GdCBT. These tools could maximize allocation of scarce resources by reducing the personnel time needed to measure fidelity, potentially freeing up more time for high-quality clinical care.

## Introduction

Digital mental health interventions (DMHIs) for common psychiatric disorders, such as anxiety, depression, and eating disorders (EDs), hold promise in alleviating the global burden of mental health challenges (Karyotaki et al., 2023). Effective mobile and online app-based DMHIs have the potential to surmount obstacles to treatment dissemination, including accessibility, cost, limited availability of professionals trained in evidence-based therapies, and stigma. Moreover, guided self-help digitally delivered cognitive-behavioral therapy (GdCBT), a form of DMHI, offers scalability, enabling a single coach to oversee more individuals than possible using a standard 1:1 model (Sasseville et al., 2023).

Such GdCBT typically integrates a supervised bachelor- or master-level individual as a coach. Coaches are trained to support the digital self-help treatment and its components as opposed to fully delivering the treatment. The coach’s role is to answer questions or provide more information that may clarify the value and execution of various digital modules and techniques, facilitate user learning, address obstacles to change, provide reinforcement, motivate continuation, personalize the intervention, and track progress (Werntz, Amado, Jasman, Ervin, & Rhodes, 2023). Coach-delivered messages offer the opportunity to gather extensive, insightful textual data to enhance comprehension of the delivered intervention and guide the improvement of forthcoming DMHIs.

As the utilization of GdCBT grows, there is a demand for advanced data analytics capabilities to assess *implementation fidelity.* Implementation fidelity in GdCBT refers to the degree to which the coaches delivered their guidance according to the trial design and theory of the digital treatment (Patterson, Rossi, Pencer, & Wozney, 2022; Waltz, Addis, Koerner, & Jacobson, 1993). Although various terms are presently used to describe this procedure, such as intervention integrity, protocol adherence, and fidelity, they all emphasize the core concept of providing the guided DMHI as developed or intended (Bellg et al., 2004; Borrelli et al., 2005). Assessment of fidelity is essentially a manipulation check that confirms that the human coach portion of the independent variable was manipulated as intended. Such fidelity provides greater confidence that the study methods were implemented well to test the question of interest and that the treatment was provided as designed (Breitenstein et al., 2010). Thus, ensuring implementation fidelity strengthens the rigor of a DMHI trial by bolstering both its internal validity (outcomes can be attributed to the treatment) and external validity (outcomes can be generalized across diverse contexts; Toomey et al., 2020). Sustained implementation fidelity aids in fine-tuning the DMHI, facilitates study reproducibility, and enables the transportability of evidence-based DMHIs to real-world practice settings.

Common components of fidelity shared between therapist-delivered therapy and GdCBT include quality or competence of intervention delivery, adherence to the treatment techniques, and not engaging in proscribed behaviors (Waltz et al., 1993). Examples of quality and adherence in guided self-help would be addressing users’ concerns and goals within the treatment model as well as answering questions about various tools, how they work, and how they are applied. Furthermore, it would include reflecting on progress, rewarding engagement, encouraging autonomy, and tailoring recommendations (Kopelovich, Buck, Tauscher, Lyon, & Ben-Zeev, 2024). Preventing prohibited targets would include not reinforcing unconstructive behaviors (e.g. avoidance).

To evaluate fidelity, an independent set of CBT-trained human coders must systematically review a substantial random sample of GdCBT coach’s asynchronous messages to users using an established rubric (Fitzsimmons-Craft et al., 2023; Ruzek et al., 2024). However, this approach to monitoring coach fidelity still runs into the same challenges as examining therapist fidelity in conventional psychotherapy. Human coder training demands extensive hours (Rodriguez-Quintana & Lewis, 2018), with additional time required to address any protocol deviations by the coders (Creed et al., 2022a, 2022b).

Natural language processing (NLP) may enhance monitoring of guided DMHI implementation fidelity. NLP is a branch of computer science that centers on acquiring, understanding, and generating common human languages, including textual data (Malgaroli, Hull, Zech, & Althoff, 2023). Advanced NLP models learn the meaning, structure, and use of language from extensive text collections, often containing billions of words via transformer-based architectures (Can et al., 2016), such as BERT (bidirectional encoder representation transformers) or GPT (generative pre-trained transformer; Ding, Lybarger, Lauscher, & Cohen, 2022). Using artificial neural networks, these models achieve a core part of deep learning, which comprises interconnecting nodes arranged in layers to perform tasks such as identifying patterns and making predictions. In traditional psychotherapy, neural networks have been used to offer insights into client-therapist interactions (Mosavi, Ribeiro, Sampaio, & Santos, 2023; Nitti, Ciavolino, Salvatore, & Gennaro, 2010; Flemotomos et al., 2021) and in GdCBT to predict process variables, such as engagement (Côté-Allard, Pham, Schultz, Nordgreen, & Torresen, 2023).

NLP, including artificial neural networks, could improve the assessment and prediction of fidelity of GdCBTs by evaluating coach-to-user message content to test adherence to CBT values, assess the frequency of positive, neutral, and negative sentiment words, and address possible aberrations from the established fidelity guidelines (Berkel et al., 2023). For example, by flagging fidelity deviation cases through NLP-automated analysis, specific feedback to coaches or supervisors could optimize clinical care (Sibley et al., 2024). Together, NLP could enhance the prediction of implementation fidelity in coach-guided DMHIs.

NLP sentiment analysis could provide additional insights regarding fidelity. Sentiment analysis entails examining patterns of positive and negative word frequencies of coach-to-user messages (Goldberg et al., 2020; Nix, Dozier, Porter, & Ayers, 2024). Such sentiment analyses might empower investigation of the tone and tenor of coach-to-user messages, including alignment with CBT principles (or lack thereof; Sadeh-Sharvit, Rego, Jefroykin, Peretz, & Kupershmidt, 2022). For instance, a study found that NLP sentiment analysis yielded an AUC of .708 to predict face to face therapist fidelity (Althoff, Clark, & Leskovec, 2016). Together, NLP tools can be used to analyze keywords, assess coach fidelity to guided DMHI protocols, provide analytics to optimize coach-to-user messages, and offer a secure place to retain and generate notes.

A recent review found 52 studies that used machine learning (ML; including NLP) to predict clinician implementation fidelity of face-to-face psychotherapies (Ahmadi et al., 2021). These ML methods performed better than random chance. Such studies typically built their ML models by capitalizing on NLP (e.g. linguistic inquiry and word count [LIWC] dictionary) (Pennebaker, Booth, Boyd, & Francis, 2015) to extract speech and related linguistic attributes from psychotherapy recordings or transcripts. Attributes are then analyzed using ML (e.g. ridge regression and maximum entropy Markov model) to predict human ratings. For example, Can et al. (2016) harnessed a Markov model with NLP attributes (e.g. contextual n-grams, meta-features, and similarities) to identify therapist reflections in motivational interviewing (MI) transcripts, attaining 73% accuracy, 93% recall (or sensitivity), and 90% specificity with human coder ratings. Mieskes and Stiegelmayr (2018) found that a holistic transcription and attributes derived from NLP and human coder ratings optimally predicted therapy session quality in patients with schizophrenia. Goldberg et al. (2020) utilized a fully automated NLP model and observed that certain therapist speech features had small yet substantial positive correlations with client-reported therapeutic alliance (Spearman’s ρ = .15, *p* < .05). NLP tools thus offered ways to analyze therapy content without heavily depending on human coders to review the fidelity quality and related metrics. It has been argued that NLP techniques could provide precise representations of human-generated codes and significantly enhance the efficiency and scope of fidelity supervision (Tanana, Hallgren, Imel, Atkins, & Srikumar, 2016).

Although multiple studies have examined fidelity in face-to-face therapies using NLP (Malgaroli et al., 2023), no study has examined ML as a means to assess fidelity to DMHIs (Ahmadi et al., 2021; Mohr, Lyon, Lattie, Reddy, & Schueller, 2017). Malgaroli et al. (2023) found that most NLP studies focused heavily on MI transcripts, with less fidelity research on other psychotherapy types. Most studies also failed to establish internal validity regarding the consistency and precision of NLP-derived fidelity monitoring systems relative to human coder ratings (Malgaroli et al., 2023; Mathur et al., 2023). Further, the best NLP and ML approaches that could model the nuances of coach-to-user messages in GdCBT have not been examined thoroughly (Berkel et al., 2023; Creed et al., 2022a). Given the increasingly prevalent adoption and utilization of DMHIs in real-world contexts, such as industry (Torous, 2023), it is imperative to devise scalable methods for assessing coach fidelity, as human assessment lacks scalability. These facts underscore the importance of examining and monitoring fidelity (as done by human coders) and determining how to make this process more scalable. Capitalizing on NLP tools might raise the efficiency and effectiveness of this labor-intensive process.

The present study thus harnessed NLP methods to evaluate their utility in automating assessment of the implementation fidelity of GdCBT coaches. Harnessing NLP and ML tools used herein could help clinical supervisors enhance the effectiveness, rigor, and quality of the supervision process. It could improve the current system where much effort is taken to both supervise coaches and train human coders to assess the fidelity of coach-to-user messages. Thus, well-performing NLP and ML algorithms that reliably classify coaches’ actions and inactions and the quality of those behaviors with good predictive accuracy might optimize the supervision time and enhance clinical care.

We examined data collected as part of a two-arm, multi-site RCT (Fitzsimmons-Craft et al., 2023), in which trained and supervised coaches supported undergraduate student users of a GdCBT program. First, we hypothesized that the coaches would show high implementation fidelity as rated by an external team of human coders. Second, we hypothesized that NLP techniques such as sentiment analysis and ML models capable of taking into account non-linearities and interactions (Polley, Rose, & van der Laan, 2011) would demonstrate good performance (with AUCs ≥ .70) in predicting coach fidelity (Haynos et al., 2021).

## Methods

### Context

Fidelity monitoring implementation was part of an extensive multi-site RCT aimed at evaluating the efficacy of a transdiagnostic GdCBT for preventing and treating anxiety, depression, or EDs among university undergraduates (Fitzsimmons-Craft et al., 2021). Undergraduates from 26 universities or colleges received an email invitation to complete screening measures. Those who met the clinical threshold or were at risk for anxiety, depression, or EDs and who were not undergoing any mental health treatment were encouraged to partake in the present RCT. Following voluntary informed consent, interested participants were randomized to receive either the SilverCloud GdCBT program (Bartholmae, Karpov, Dod, & Dodani, 2023; Fitzsimmons-Craft et al., 2023; Laboe et al., 2024; Richards et al., 2018) or referral information to mental healthcare treatment options offered within their university.

### Coaches

Coaches (*n* = 73) served as guides to the 6-month SilverCloud GdCBT program. They had a modal age of 20–29 years (46%) instead of older age groups (30–39 years: 35%; 40–50 years: 11%; 51+ years: 8%). Most were women (81%) compared to men (16%) and other gender identities (3%). Regarding race, most were White (non-Hispanic; 57%), followed by Asian (24%), did not respond (11%), more than one race (5%), and African American (3%). Regarding clinician/trainee status, none of the coaches were undergraduate students. Most were MA students, followed by doctoral students, and others who were working adults with at least a B.A. degree with an interest in volunteering. Ph.D.-level clinical psychologists trained coaches to understand the core principles of CBT, i.e. what it is, how it works, change mechanisms, and examples of how to articulate CBT principles. The coaches also received extensive standardized training on digital coaching and asynchronous messaging and attained familiarity with the SilverCloud GdCBT program. The coaches also met weekly with a supervisor. Please see Fitzsimmons-Craft et al. (2023) for additional information on coach training.

### Coders

Sixteen undergraduate research assistants served as coders who rated the quality of coach fidelity. Coders represented a separate group from coaches. They attended weekly meetings to learn about CBT, what it is, how it works, and why it is effective through assigned readings, didactics (Tolin, 2016), and weekly discussions. Ph.D. candidates trained the coders and facilitated these didactics. For instance, coders were taught how CBT differed from supportive psychotherapy (Moncher & Prinz, 1991) and to identify when coaches wrote messages that deviated from CBT principles by enabling avoidance patterns, self-sabotaging, or other emotionally driven behaviors. Simultaneously, coders learned how to detect when coaches gave appropriate encouragement to engage with skills taught by the GdCBT program. Further, the lead author created standardized training videos (63 minutes total) on how to review and rate coaches’ messages on implementation fidelity. For example, the degree to which the coaches adhered to the treatment protocol by properly prescribing targets and avoiding any of the prohibited or proscribed targets (Waltz et al., 1993). Coders met with clinical supervisors weekly to discuss and resolve any assigned rating discrepancies. Moreover, coder ratings were regularly checked by then-Ph.D. candidates (GNR and NHZ) with at least three years of CBT practicum training by a licensed Ph.D.-level clinical psychologist (MGN). These coder ratings were also used as feedback during the coach training and supervision process.

### Users

In the two-arm RCT (Fitzsimmons-Craft et al., 2021), 3,381 participants with anxiety, depression, or EDs (with a particular focus on bulimia nervosa and binge ED) were randomly assigned and enrolled in SilverCloud GdCBT at baseline, referred to as “users”. Each user was assigned a coach. On average, users were 20.2 years old (*SD* = 4.03, range = 18–58). Regarding sex assigned at birth, 73.1% were female, 26.7% were male, and the remaining 0.2% were intersex. Concerning race, 64.2% were White, followed by Asian (14.5%), African American (7.22%), Multiracial (6.54%), American Indian or Alaskan Native (0.8%), and Native Hawaiian or Pacific Islander individuals (0.3%). Regarding ethnicity, 82.2% identified as non-Hispanic, 17.4% were Hispanic, and the remaining 0.4% did not disclose.

### Overview of GdCBT

SilverCloud was a scientifically backed GdCBT program for anxiety, depression, and EDs (Benjet et al., 2023; Fitzsimmons-Craft et al., 2020; Taylor, Graham, Flatt, Waldherr, & Fitzsimmons-Craft, 2021). It provided six to eight primary modules for a specific mental health issue, each taking approximately 20 minutes to complete. The modules contained instructional psychoeducation, quizzes, interactive practices, vignettes, and videos. Access to the program spanned six months.

### Procedures

Coaches were instructed to send asynchronous messages to users twice weekly during the first two weeks and then once a week from the third week onward. Users were encouraged to engage with weekly lessons (called modules) and had the option to message coaches for clarification on therapy concepts or additional support. Coaches reviewed all user activity and any messages users sent to them. Ph.D.-level clinical psychology supervisors (EEF, ER, CBT, and MGN) taught them how to respond to those messages in ways consistent with CBT principles.

A brief rubric was developed to instruct coders in rating the degree to which each coach adhered to best practices, i.e. did what they were expected to do. Specifically, the outcome of interest in the present study was a consistent coder rating of “yes” instead of “no”, implying that the coach displayed exemplary supportive accountability behaviors (Mohr, Cuijpers, & Lehman, 2011). Examples included managing and reviewing users’ lessons, showing genuine interest in the user as a person, assisting in clarifying and specifying users’ primary concerns or goals, and not reinforcing negative (including self-sabotaging) behavior or mindsets (refer to Supplementary Table S1 for details on the coaching implementation fidelity best practices rubric). The rubric was developed based on best recommendations for GdCBT where the coach personalized therapy skill provision while aiming to ensure that each statement written in the coach-to-user message aligned with CBT principles and theories (Kendall et al., 2023; Thew, Rozental, & Hadjistavropoulos, 2022). A randomly selected subgroup was coded (Richards et al., 2018).

A randomizer was created so that coders would rate 15–20% of any one coach’s messages to users every month, which translated to about 560 messages per week. Two coders independently rated all randomly selected coach-to-user messages. Discrepancies in ratings were resolved as far as possible during weekly meetings. Coder ratings were also regularly checked by Ph.D. candidates with at least three years of CBT practicum training by licensed Ph.D.-level clinical psychologists. This concurred with established implementation fidelity practices in face-to-face CBT (Waltz et al., 1993) to minimize “therapist drift” (deviation from the intended protocol; Speers, Bhullar, Cosh, & Wootton, 2022). Supplementary Table S2 offers real examples of coach-to-user messages or utterances pertinent to each fidelity code.

Relatedly, user-to-coach messages were excluded. Only coach-to-user messages were examined, as these messages spoke squarely about the coach’s fidelity to delivering the GdCBT based on CBT principles and the coach’s capacity to implement such content to offer appropriate guidance (Bernstein et al., 2022). Coach-to-user messages directly indicated the coach’s provision of CBT components, individualized feedback, and attempts to initiate and sustain engagement, all of which were vital in the effective delivery of GdCBT (Meyer, Wisniewski, & Torous, 2022; Myers et al., 2024).

### Data analyses step 1: Inter-rater agreement among human coders

**Figure 1.**
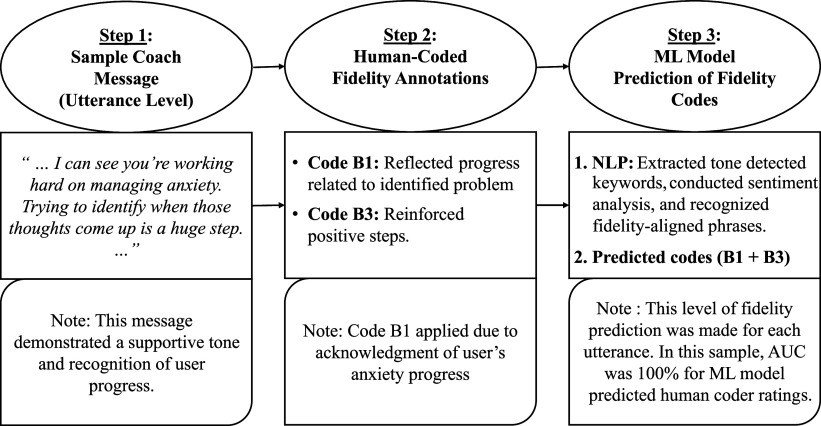
Schematic diagram of data analytic steps. *Note.* ML, machine learning; NLP, natural language processing. Please refer to Supplementary Table S1 for more information on the best coach fidelity rubric codes.

Figure 1 offers a schematic diagram of the data analytic steps. Inter-rater agreement between two coders was indexed with the intraclass correlation coefficient (ICC), weighted Cohen’s kappa (κ), and percentage (%) of agreement (Shrout & Fleiss, 1979). We calculated ICC using a 2-way random-effects model and κ values with the *irr R* package (Gamer, Lemon, Fellows, & Singh, 2007).

### Data analyses step 2: Natural language processing (NLP) sentiment analyses

We employed cutting-edge ML, including NLP, from Scikit-learn, an open-source Python-based library that supports supervised, semi-supervised, and unsupervised ML (Pedregosa et al., 2011). Three *R* packages – *textrecipes* (Hvitfeldt, 2023), *tidyverse* (Wickham et al., 2019), and *tidytext* (Silge & Robinson, 2016) – were also used to conduct NLP on all coach-to-user message data. The following steps were taken for abstracting data. An interdisciplinary team comprising clinicians, computer scientists, and statisticians carried out these procedures.

**Feature engineering.** Regardless of the model used, all coach-to-user message data was converted to a numerical format and then partitioned into training and test sets using tenfold nested cross-validation (10F-CV) with the *nestedcv* (Lewis et al., 2023) *R* package to prevent data leakage and overfitting (i.e. inadequate external validity or generalizability; Degtiar & Rose, 2023). We extracted all words from coach-to-user messages for a total of 13,529 coach-to-user messages that human coders rated. All coach messages were organized into one token (i.e., a meaningful unit of analysis) per row to compute word frequencies to comprehend the tone and content, including the degree of consistency with CBT principles, and to conduct sentiment analysis (Liu, 2012).

**Sentiment analysis.** Sentiment analysis, a subcategory of NLP (Eberhardt et al., 2024), was carried out by counting the positive and negative sentiment words across all coach-to-user messages using three established emotion word dictionaries (or sentiment lexicons): Bing (Liu, 2012), AFINN (Nielsen, 2011), and NRC (Mohammad & Turney, 2012). In particular, it examined the extent to which coaches used positive sentiment words, such as “change”, “use”, “exercise”, and “practice”, as well as emotional words (e.g. anger, anticipation, disgust, fear, joy, sadness, surprise, and trust) that conveyed more or less encouraging or supportive tone. It examined whether the frequencies of various sentiment words were important in predicting coach fidelity. By studying the sentiment of coach-to-user messages, we were able to detect whether sentiment word associations and patterns could inform optimal practices for preserving GdCBT integrity (Mieskes & Stiegelmayr, 2018; Provoost, Ruwaard, van Breda, Riper, & Bosse, 2019). The following step details the models used in harnessing sentiment analysis to predict coach fidelity.

### Data analyses step 3: Predictive ML modeling to automate the evaluation of coach fidelity

Supervised ML methods (Becker et al., 2018) were employed to test the degree to which abstracted coach-to-user message features reliably predicted human coders’ fidelity ratings. It examined whether the coaches consistently achieved their aims at the outset, intermittently, and during most review sessions. The binary outcome was dummy coded (1 = *met fidelity* versus 0 = *did not meet fidelity*). We rigorously assessed various ML algorithms to determine their suitability for predicting optimal (versus non-optimal) coach behaviors, guided by CBT theories (Funk et al., 2020). The bias-variance trade-off (Geman, Bienenstock, & Doursat, 1992; Hastie, Tibshirani, & Friedman, 2009) underscored the delicate balance in model selection. Each ML algorithm assessed the ability to predict coach fidelity via nested 10F-CV (Genuer, Poggi, & Tuleau-Malot, 2010; Varma & Simon, 2006).

We used the Super Learner method to build predictive ML models for detecting coach fidelity (van der Laan, Polley, & Hubbard, 2007). Super Learner is an ensemble algorithm employing a stacking process to discern the optimal weighted amalgamation of various ML algorithms using nested CV to minimize the loss function’s value (Polley et al., 2011). Super Learner’s advantage lies in its capacity to incorporate a diverse optimal weighted array of predictive ML models, often matching or surpassing the top-performing base algorithm (Naimi & Balzer, 2018). We harnessed 11 base algorithms to construct the Super Learner. These included Gaussian Naive Bayes, K-Nearest Neighbors, Logistic Regression, Multilayer Perceptron, Decision Tree, Ada Boost, Bagging, Random Forest, Extra Trees, Support Vector Machine, and Super Learner. See online supplemental materials (OSM) for more details on evaluated models. We reported the results of the Super Learner method as well as each individual ML algorithm.

All NLP variables were incorporated for each predictive model without employing any feature selection procedures. We used a nested CV approach to mitigate the optimistic bias in non-nested CV procedures, where the same dataset was used for both hyperparameter tuning and model selection, leading to information leakage and bias (Lewis et al., 2023). Specifically, we performed two 10F-CV loops: the inner CV loops for hyperparameter tuning and the outer CV loops for model evaluation, comparison, and selection (Cawley & Talbot, 2010). Prediction performance was assessed via receiver operating characteristic (ROC) analysis, with the area under the ROC curve (AUC) as the evaluation metric. Sensitivity, specificity, positive predictive value (PPV), and negative predictive value (NPV) values were also computed (Pepe, 2003).

## Results

### Step 1: Inter-rater agreement among human coders about coach fidelity

ICC between raters ranged from .980–.992, κ = .894–1.000, and percentage (%)-agreement = 97.4–100.0. Thus, excellent inter-rater reliability among the human coders indicated consistency in ratings of coach fidelity. Table 1 offers examples of high-fidelity coach-to-user messages, whereas Table 2 presents instances of low-fidelity (or suboptimal) ones. Further, the coders assessed whether coaches fulfilled the compulsory fidelity targets at the beginning of the intervention (Supplementary Table S1 Criteria A score: *M* = 3.35 out of 4, *SD* = 0.33) and for most review sessions (Criteria B score: *M* = 3.12 out of 4, *SD* = 0.43). Coaches also consistently fulfilled at least 3 of 8 optional behavioral targets when writing each review message (Criteria C score: *M* = 3.10 out of 8, *SD* = 1.08). Last, the coaches generally avoided proscribed targets, as reflected by the low scores (Criteria D score: *M* = 0.01 out of 4, *SD* = 0.10). Our first hypothesis was thus fully supported.

**Table 1.** Examples of high coach-to-user implementation fidelity messages

#	De-identified coach-to-user message
1	It’s great to hear from you. I’m glad that you took some time to practice the skills on your own. That helps the information stick and become more applicable to your life. The positive thoughts journal and general mindful approach to that exercise sounds like a wonderful tool for your mental health. When it comes to your negative thoughts, are there any that you have been struggling with more so than others? It may be a great time reflect on these and practice counterbalancing them with the Core Belief tool. I hope to see you back here next week! I’ll be in touch with your review on [date]!
2	I hope you are doing well! When you signed up for the iAIM EDU study four weeks ago, you shared that you wanted to work on your anxiety and that you hoped the program would help you make some changes. However, it seems like it has been difficult for you to engage with the program. What would you think about trying to focus on something different? I have loaded the Boosting Behavior module into your program. One of the goals of this module is to look at how our behaviors can impact our moods! Please feel free to take a look at this new module or the other modules you have available, and let me know if you have any questions. I will check in with you next week!
3	How are you feeling this week? Thank you again for messaging me last week about everything that is going on in your life. Did you make any decisions? I hope you were able to use some of the tools you have been learning in SilverCloud and also the relaxation techniques that are so important – that you mentioned helped you. I sent you the Managing Worry module last week. I hope you get a chance to use it this week. Also, you were last on Facing Your Fears. Keep going – you are doing SO well! Message me if you get a chance and let me know how you are and what you are focusing on. I will check in with you again next [date]. Like I said last week – I know you can get through this hard time and use the techniques you are learning to help you through it. Stay well [User]!
4	Great job this week with SilverCloud! You logged in once and worked through 2 modules, Welcome to SilverCloud and Getting Started. WOW – awesome awesome work! Great job using the tools section and thank you for sharing them with me. Your larger goal of feeling better by controlling your anxiety is a very positive and doable goal. We can keep that at the front and center as you work through these modules and learn more about how you are feeling, the thoughts/feelings/behavior cycle, and what can be done to boost your positive feelings about yourself. I really like the smaller goals you have set for yourself of doing a breathing exercise daily and exercising daily. Can you tell me what impact the breathing and exercise will have on you on a daily basis? And do you have someone that you are with that can help you be accountable for these goals? Lastly, let me know if you were able to accomplish these 2 goals this week and how it impacted your days. I cannot wait to hear about it! Next you will start on Understanding Feelings. You will learn about difficult emotions and how we all can have physical reactions to our emotions. I think this will be a really helpful initial step for you in working toward improving things. I’m so glad you are aware of your physical body sensations when you have anxiety, like your heart pounding and the choking feeling. Knowing how you feel physically is a very good thing to be aware of. With time and working through the program I have confidence you can decrease these feelings. I wanted to let you know that you have the option of setting up a 5–15 minute phone chat with me in the next week or so. This would be done through a conference line I provide. The purpose of this would be to help me get to know you better and learn more about your goals for this program. I’d love to hear about why you are interested in SilverCloud and answer any questions you might have. Please let me know if you are available for a 15-minute call next [date] or anytime after that. Our next review will be [date]. Good luck this week and I’ll be in touch next [date]. Take care of yourself and give yourself a pat on the back for doing such great work in SilverCloud.:)
5	Great job going through the program at a pace that feels comfortable for you and for a variety of tools. Remember to apply any helpful information about managing and understanding your depression concerns to your life, this way the program can become personalized. I see that you would like to replace your unhealthy habits with more healthy and goal-directed behaviors. The Understanding Feelings module is a great place to begin making the connection between thoughts, feelings, and behaviors so that you may feel more prepared to handle difficult emotions as they come up. I also recommend you try the Goal Setting tool, which is useful for breaking down bigger goals into small manageable steps. Best of luck as you get started with this and other modules which are all aimed at improving your mental health and helping you achieve your goals! On a final note, I wanted to let you know that you have the option of setting up a 15 minutes phone chat with me in the next week or so. This would be done through a conference line I provide, so there is no need to share your private phone number with me. The purpose of this would be to help me get to know you better and learn more about your goals for this program. I’d love to hear about why you are interested in SilverCloud and answer any questions you might have. Please let me know if you are available for a 15-minute call during the following times: [Dates]. The phone call is an opportunity for us to get to know one another and for me to understand what you hope to get out of SilverCloud and how I can best support you. I’ll be in touch for your next review on Wednesday! Good luck moving onto Understanding Feelings and Boosting Behaviors modules. I look forward to hearing from you!

*Note.* These examples showed that these coach-to-user messages successfully implemented some of the following fidelity targets (refer to Supplementary Table S1 in the OSM): encouraging autonomy, reflecting progress, using open-ended questions, reinforcing positive steps, providing personalized recommendations, and supporting ongoing engagement.

**Table 2.** Examples of suboptimal coach-to-user implementation fidelity messages

#	De-identified coach-to-user message
1	Just checking in for your weekly review! I know you have not had a chance to complete any modules this last week. That is totally okay! You can come back and check it out at your convenience. Next week, I will check in to see if you have had a chance to complete anything on Silvercloud on [date]!
2	This is your fourth review as part of the iAIM study, so I will be switching to weekly reviews. I hope you are able to log back in soon and explore Silvercloud. I will flag a great place to get started at your convenience when you find the time. It’s a module that covers worry and anxiety! As a reminder, the program will be available to you for six months, and you can check it out at your leisure. Your next review will be on [date]!
3	I have not seen you online for a while. Please log back in and continue checking out the helpful content. There is a lot of good information to help you with symptoms of anxiety and/or depression. I will check in again with you in a month.
4	Just a reminder that you can reach out to me if you are having any difficulties using the program or want to provide any feedback about it. Feel free to message away!
5	How was your week? First I would like to apologize for some connectivity issues I had yesterday and I could not send out your review. Second I want to point out to you the significant chance in your chart! I also want to emphasize that this is the result of the efforts you have been putting towards your efforts. It is fair to take a week off;) As1 suggestion, keep working on your program to keep consistency and I will reach out to you next Friday!

*Note.* These examples showed that these coach-to-user messages failed to implement some of the following fidelity targets (refer to Supplementary Table S1 in the OSM): clarifying user roles or expectations, demonstrating personalization, supporting or encouraging autonomy, reflecting user progress or positive steps, and using open-ended questions.

### Step 2: Natural language processing (NLP) sentiment analyses

**Figure 2.**
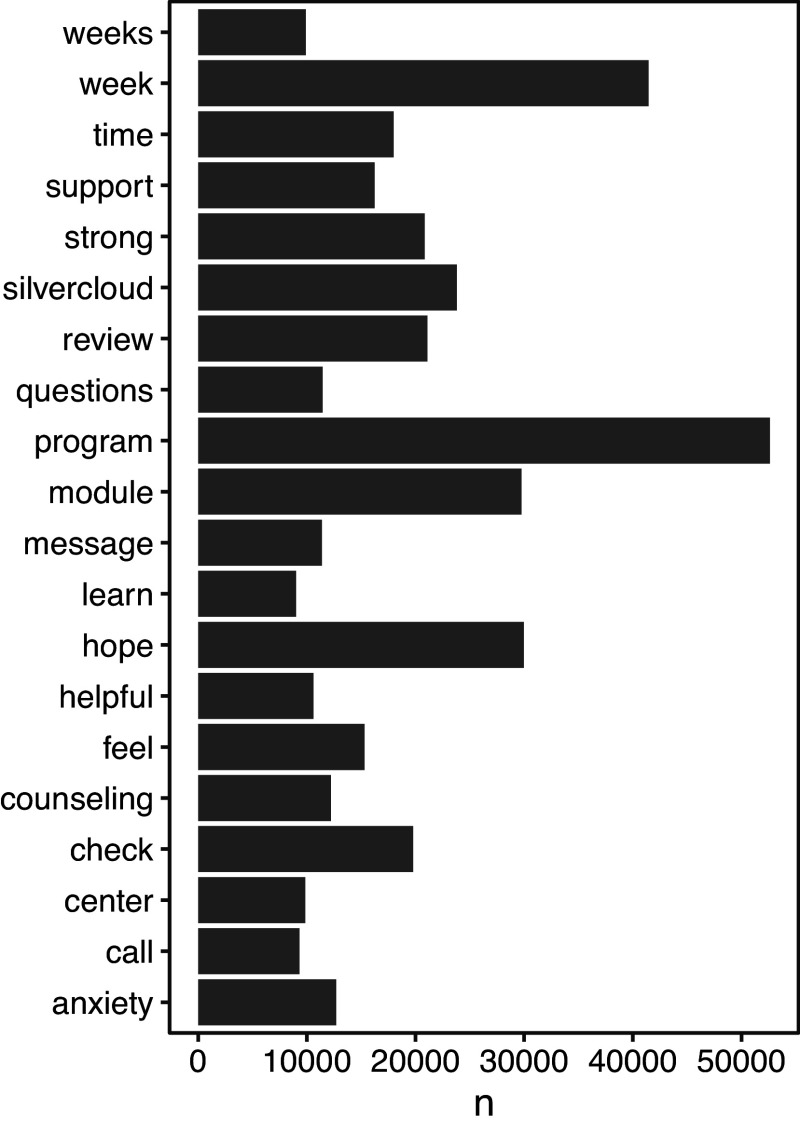
Top 20 most frequently used words by GdCBT coaches when writing messages to users. *Note.* GdCBT, digital cognitive-behavioral therapy, n, frequency (word count).

**Figure 3.**
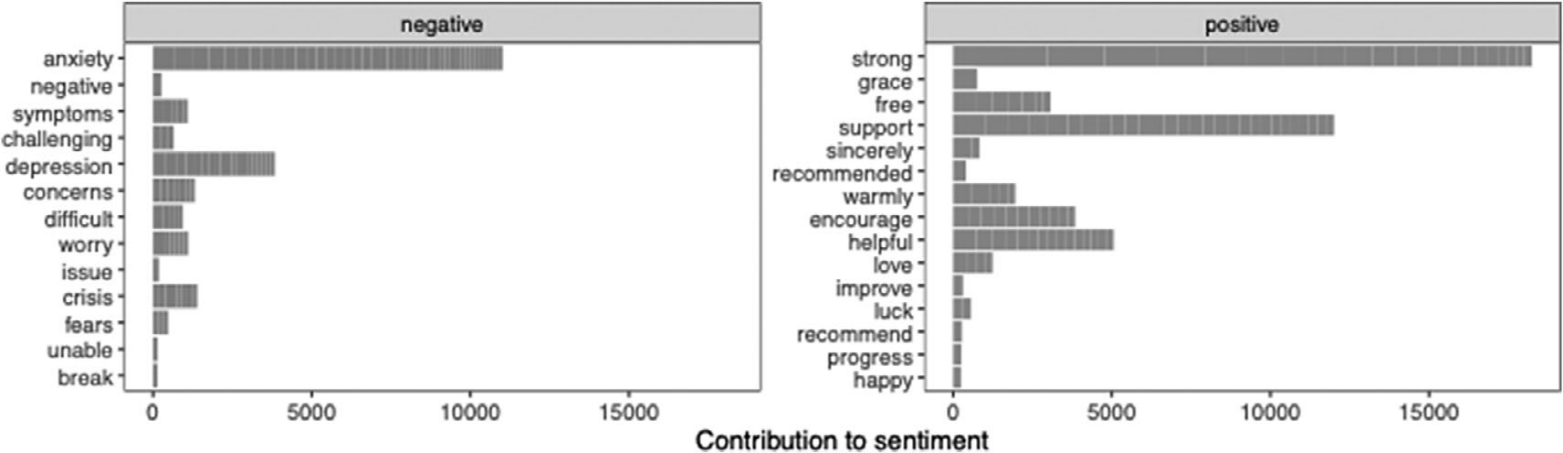
Frequency of emotion sentiment words using the Bing sentiment lexicon.

Figure 2 shows the 20 most frequently used sentiment words in coach-to-user messages. Sentiment words were classified with high consistency across all word lexicons (*r* values = .943 to .994), as depicted by the correlation matrix in Supplementary Table S3. Moreover, Figure 3 displays the most common positive (e.g. “helpful”, “strong”, and “support”) and negative (e.g. “anxiety”, “depression”, and “symptoms”) sentiment words. Using the Bing lexicon, sentiment word counts were slightly more positive (*n* = 7,792, 53.7%) than negative (*n* = 6,710, 46.3%). Using the NRC lexicon, positive sentiments comprised the highest word counts (*n* = 12,275, 26.3%), followed by these words: “trust” (*n* = 6,687, 14.3%), a negative sentiment word (*n* = 5,913, 12.7%), “anticipation” (*n* = 4,961, 10.6%), “joy” (*n* = 4,300, 9.2%), “fear” (*n* = 3,405, 7.3%), “sadness” (*n* = 3,306, 7.1%), “anger” (*n* = 2,499, 5.4%), “surprise” (*n* = 2,036, 4.4%), and “disgust” (*n* = 1,303, 2.8%). Using the AFINN lexicon, the ratio of positive to negative sentiment words was 4.1.

Following statistical recommendations to facilitate interpretation (Iacobucci, Posavac, Kardes, Schneider, & Popovich, 2015), the ordinal fidelity score summed across all domains was binarized to predict above versus below median implementation fidelity. Higher scores indicated better fidelity. All sentiment words extracted from all lexicons had good classification accuracy in predicting coach fidelity across all ML classifiers (Table 3). The best-performing classifiers were Extra Trees (74.48%), Decision Trees (74.41%), and Ada Boost (74.31%). The best-performing Extra Trees classifier had a sensitivity (true positive rate) of 69.54%, specificity (true negative rate) of 66.05%, positive predictive value (PPV; precision) of 67.26%, and negative predictive value (NPV) of 68.37%. Table 4 offers an interpretive summary.

**Table 3.** ML predictive performance of sentiment analyses with NLP to predict unique coach implementation fidelity

Classifier	AUC
Gaussian Naive Bayes	72.961%
K-nearest neighbors	73.507%
Logistic regression	73.497%
Multilayer perceptron	73.866%
Decision trees	74.411%
Ada boost	74.308%
Bagging	73.509%
Random forest	73.455%
Extra trees	74.475%
Support vector machine	73.883%
Super learner	73.897%

*Note.* ML, machine learning; NLP, natural language processing; and AUC, area under the receiver operating characteristic curve.

**Table 4.** Interpretation of performance metrics in predicting coach implementation fidelity

Metric	Definition
AUC	AUC represents the likelihood that the model assigns a higher rank to a random case of meeting fidelity compared to a random case of not meeting fidelity. An AUC of 0.5 signifies prediction no better than chance level. AUC values between 0.50 and 0.70 indicate poor prediction, values from 0.70 to 0.80 represent acceptable prediction, values from 0.80 to 0.90 denote excellent prediction, and values above 0.90 indicate outstanding prediction.
Sensitivity	Sensitivity quantifies the proportion of true positive cases correctly identified by the model as meeting fidelity criteria. It assesses how well the model detects cases where coaches adhere to prescribed standards of d-CBT implementation among all cases that actually meet fidelity. It emphasizes the model’s ability to minimize false negatives, which are cases inaccurately classified as not meeting fidelity standards when they actually do.
Specificity	Specificity quantifies the proportion of true negative cases correctly identified by the model as not meeting fidelity criteria. It assesses how well the model distinguishes cases where coaches did not adhere to prescribed standards of d-CBT implementation among all cases that actually do not meet fidelity. It emphasizes the model’s ability to minimize false positives, which are cases inaccurately classified as meeting fidelity standards when they actually do not.
PPV	PPV indicates the likelihood that cases predicted by the model as meeting fidelity criteria actually do meet those criteria. PPV denotes how well the model identifies true cases of fidelity adherence among all cases predicted to meet fidelity standards.
NPV	NPV indicates the likelihood that cases predicted by the model as not meeting fidelity criteria actually do not meet those criteria. NPV reflects how well the model identifies true cases of non-fidelity (instances where fidelity is not met) among all cases predicted not to meet fidelity standards.

*Note.* AUC, area under the receiver operating characteristic curve; d-CBT, digital cognitive-behavioral therapy; PPV, positive predictive value; NPV, negative predictive value.

### Step 3: Predictive ML modeling to automate the evaluation of coach fidelity

Table 5 presents the model performance of various classifiers. Based on AUC values, the three worst-performing ML candidate algorithms were Gaussian Naive Bayes (62.97%), Ada Boost (68.83%), and K-nearest neighbors (71.56%). Conversely, the three best-performing ML candidate algorithms were Support Vector Machine (75.14%), Extra Trees (75.614%), and Super Learner (76.06%). The Super Learner thus had a 76.06% likelihood of correctly ranking a randomly chosen case example (i.e. meeting fidelity) higher than a randomly chosen non-case example (i.e. not meeting fidelity). The final Super Learner model achieved sensitivity of 57.04%, specificity of 86.69%, PPV of 71.70%, and NPV of 77.35%. Our second hypothesis was supported.

**Table 5.** Model performance of various classifiers to automate the evaluation of coach implementation fidelity

Classifier	AUC
Gaussian Naive Bayes	62.966%
K-nearest neighbors	71.560%
Logistic regression	71.635%
Multilayer perceptron	72.159%
Decision trees	73.382%
Ada boost	68.829%
Bagging	74.292%
Random forest	73.831%
Extra trees	75.614%
Support vector machine	75.140%
Super learner	76.063%

*Note.* AUC, area under the receiver operating characteristic curve.

## Discussion

Prior DMHI studies have evaluated whether regular therapist-to-user emails adhered to prescribed behaviors using human coders (e.g. Hadjistavropoulos, Schneider, Klassen, Dear, & Titov, 2018). We built on such work by testing the promises and shortcomings of ML and NLP (including sentiment analysis) to automate fidelity monitoring in GdCBT. Consistent with expectations, coaches who underwent intensive training and supervision during a meticulously monitored, multi-site RCT (Fitzsimmons-Craft et al., 2021) showed good implementation fidelity as rated by well-trained and monitored human coders.

Coders rated coaches’ GdCBT messages for alignment with CBT theory to promote skill usage (e.g. exposure therapy) and avoid prohibited actions (e.g. enabling avoidance). Notably, our observed coder inter-rater agreement (.894–1.000) using diverse metrics (ICC, κ, and %-agreement) fell within ranges considered to be excellent (Cicchetti, 1994). Our coder ratings were also notably higher than other empirical studies documented by a recent psychotherapy fidelity review (κs = .24–.66; Ahmadi et al., 2021). The excellent inter-rater reliability might be partly because our fidelity rubric had an optimal number of codes (20 codes in total), and all codes were more concrete than abstract. In the psychotherapy fidelity literature, the number of codes varied from two (Xiao, Imel, Georgiou, Atkins, & Narayanan, 2015) to 209 (Gaut, Steyvers, Imel, Atkins, & Smyth, 2017). Fewer codes corresponded to improved inter-rater agreement and predictive performance, and the reverse was also true (Ahmadi et al., 2021). Concrete codes, such as ours, were predicted more accurately than abstract, conceptual codes. Our concrete codes covered four classes: targets for the start of the GdCBT (four codes), compulsory targets for most GdCBT sessions (four codes), optional targets for some sessions (eight codes), and proscribed behaviors (four codes). Examples of concrete codes included “Demonstrates interest in the user as a person” and “Reinforces positive steps (even if miniscule)”. Conversely, other fidelity coding systems, such as the 19-code Motivational Interviewing Skill Code (MISC) and 10-code Motivational Interviewing Treatment Integrity (MITI), comprised more abstract codes (e.g. advising, confrontation, and emphasizing autonomy; Atkins, Steyvers, Imel, & Smyth, 2014; Imel, Steyvers, & Atkins, 2015; Tanana et al., 2016; refer to Supplementary Tables S4 and S5 for more details on these alternative psychotherapy fidelity coding scales). Our fidelity rubric also overlapped somewhat with face-to-face CBT rubrics, such as the 30-code ACE Treatment Integrity Measure (ATIM; Bendall et al., 2015), 25-code Cognitive Therapy Adherence and Competence Scale (CTACS; Barber, Liese, & Abrams, 2003), 14-code Cognitive Therapy Scale-Revised (CTS-R; Blackburn et al., 2001), and 88-code Cognitive Processing Therapy (CPT)–Therapist Adherence and Competence Protocol (Marques et al., 2019; refer to Supplementary Tables S6–S10 for summaries of alternative CBT fidelity measures). These fidelity rubrics garnered mean ICC values that ranged between .57 and .95, with values falling mainly within the .60–.80 range. Despite some overlap between face-to-face CBT and GdCBT, such as feedback, reinforcement, and tailoring to the individual, inter-rater agreement differences between the present and prior studies could also be due to codes specific to GdCBT, such as “Encourages use of SilverCloud”. In addition, compared to briefer rubrics (e.g. the two-code system utilized by Xiao et al., 2015), our 20-code rubric probably enabled a more nuanced evaluation while maintaining concrete instructions sufficient for high agreement. Together, the thorough and concrete, CBT-focused feature of our GdCBT fidelity coding system and high-intensity coder training probably led to the high observed inter-rater reliability.

Further, coach-to-user messages comprised more positive (e.g. “helpful” and “support”) than negative sentiment words (e.g. “anxiety” and “depression”). If replicated, this outcome might indicate that a skew toward more positive than negative sentiment words in coach-to-user communications is integral to ensuring consistency with CBT principles in addition to enhancing therapy conversations (Pérez-Rosas et al., 2018). Ongoing research on this phenomenon would further clarify the value of NLP sentiment analysis in maintaining treatment integrity for GdCBT.

Overall, our best-performing predictive ML models achieved acceptable performance metrics (AUC = 75–76%, sensitivity = 57–70%, specificity = 66–87%, PPV = 67–72%, NPV = 68–77%). The optimal models were Extra Trees and Super Learner. Extra Trees, an amalgamation of decision trees that included more randomization in the decision rule development process than AdaBoost, Decision Trees, and Random Forests, possibly decreased variance and enhanced generalizability of the predictive models (Geurts, Ernst, & Wehenkel, 2006). Super Learner is a stacked ensemble ML method that combines predictions from multiple base classifiers using cross-validation to optimize weights and reduce prediction error (van der Laan et al., 2007). These ensemble techniques’ stronger rigor and ability to manage complex, non-linear associations likely explained their greater predictive power than other single-component ML algorithms examined herein. Other simpler algorithms (Gaussian Naïve Bayes, Logistic Regression, and K-Nearest Neighbors) likely performed worse with fidelity data due to their strong assumptions about linearity and predictor independence and lack of scalability to big datasets (Beyer, Goldstein, Ramakrishnan, & Shaft, 1999; Ng & Jordan, 2001). Support Vector Machine might not have performed well due to its sensitivity to hyperparameter tuning (Steinwart, 2008). In addition, other NLP studies using ML to predict therapist fidelity for face-to-face psychotherapy yielded comparable quality when predicting human coder-rated scores, as reflected by AUCs of .72–.79 (Atkins et al., 2014; Can et al., 2016; Gallo et al., 2015; Gaut et al., 2017). Considerable discussion exists about the most suitable metric in general (Handelman et al., 2019) and specifically in the context of implementation fidelity (Ahmadi et al., 2021). Based on statistical (Hernández-Orallo, Flach, & Ferri, 2012) and practical (Ahmadi et al., 2021) considerations, AUC would take precedence over other metrics for predicting fidelity since it considers the balance between true positive rate and true negative rate. Consistent with this, we found that coaches more often coded for prescribed behaviors than proscribed ones.

Moreover, our study built on prior fidelity studies that utilized ML by testing the generalizability of predictive models to unseen data (i.e. a new context; Ahmadi et al., 2021) using the nested CV approach that prevents data leakage (Lewis et al., 2023). We could only locate a single study that capitalized on ML for motivational interviewing transcripts and tested it on unseen data (Idalski Carcone et al., 2019). Akin to our results, their ML models attained an acceptable degree of concordance with human coder ratings. Data-driven insights might thus enhance clinical supervision and practice by equipping coaches with evidence-based communication techniques to better customize their asynchronous interactions with users. Collectively, automated fidelity coding could be a vital initial step toward achieving effective guided DMHIs that require minimal to no human coders.

From a clinical practice perspective, the present study offers a step toward actionable solutions for enhancing the implementation fidelity of DMHIs. Nevertheless, the necessity for coaches undermines a frequently advocated benefit of DMHIs: their scalability, which involves effortless deployment to diverse global populations with varying economic and ethnic attributes needing good mental healthcare (Hollis et al., 2017). Simultaneously, growing calls for adopting task-sharing approaches (i.e. deploying persons without rigorous background training in psychological theories and techniques as coaches for guided DMHIs; Barnett, Puffer, Ng, & Jaguga, 2023) should not compromise fidelity quality. Eliminating the need for human coders to monitor and maintain fidelity by using sophisticated NLP and ML tools, will provide clinical supervisors deploying scalable DMHIs with more staffing and the ability to re-allocate scarce human resources to serve as human coaches instead.

The current NLP analysis intentionally did not test the association between fidelity and DMHI outcomes because doing so was tangential to the study aims and a separate research question (Malgaroli et al., 2023; Perepletchikova, 2005). Treatment fidelity or integrity – the extent to which a GdCBT coach abided by CBT theories, principles, and planned treatment delivery – was essentially a manipulation check of the internal validity of the coach portion of the GdCBT (Breitenstein et al., 2010; Perepletchikova, 2005). Methodologists recommend that manipulation check assessments are discriminated theoretically and procedurally from the outcome (Kazdin, 2021). This aim was achieved using reliable human coders of coach-delivered intervention text as our dependent measure, which is the suggested method of fidelity assessment (Kazdin, 2017; Vallis, Shaw, & Dobson, 1986; Waltz et al., 1993). Thus, we focused on testing ML prediction of predetermined gold-standard fidelity metrics, retaining a discrete boundary between fidelity assessment and DMHI outcome evaluation, which aligned with best practice methodological recommendations (Kazdin, 2021). The question of interest was whether it was possible to forego time-consuming human ratings and instead use an ML algorithm to determine if coaches delivered their messaging with fidelity. Importantly, if fidelity (or ML prediction of fidelity) were associated with outcome, it would not confirm that the ML algorithm predicted coach fidelity. Therefore, studying the relationship between coach fidelity and DMHI outcomes could be a separate direction in the future.

The present study had several limitations. First, as our analyses aggregated coach fidelity ratings throughout the intervention course, future studies on guided DMHIs should examine how fidelity evolves over time using methods that account for the longitudinal data structure of fidelity. Second, DMHIs frequently lack the interactivity of in-person psychotherapy or teletherapy that offers non-verbal cues, as they frequently depend on asynchronous communication and user engagement. On that note, future DMHI studies should determine the value of user-to-coach messages in studying coach fidelity, given that those were excluded from the present analysis but could offer insights about the quality of help users received. Third, future research should compute weighted log-odds ratios or similar metrics for sentiment analysis since specific negative or positive sentiment words (e.g. “terrible” and “excellent”) might yield a more substantial effect than others (e.g. “unhelpful” and “nice”). Fourth, NLP approaches might struggle with comprehending context, implicit meanings, and sarcasm in clinical conversations. Thus, sentiment analysis, although insightful, might not inform the complex nature of therapeutic DMHI conversations. Despite these limitations, the study’s strengths included the novelty and pragmatism of our research question and approach to optimizing the delivery of GdCBTs. Relatedly, our study filled an essential knowledge gap in GdCBT research and practice since a key challenge in assessing coaching effectiveness is the frequent dearth of reporting on implementation fidelity, training protocols, and unique coaching outcomes (Meyer et al., 2022).

To conclude, NLP and ML methods (especially SuperLearner and Extra Trees) were dependable approaches for monitoring coach fidelity in guided GdCBT. Most DMHI studies do not examine their fidelity (Schueller & Torous, 2020). When fidelity was examined, there was reliance on human coders. This can be burdensome and may not be scalable to a clinically meaningful level in a time-efficient fashion. This resource-intensive procedure necessitates plausible NLP and ML solutions. Building on current and existing work (Nook, Hull, Nock, & Somerville, 2022), future research endeavors should leverage NLP and ML techniques with coach-to-user messages and related textual data to predict guided DMHI outcomes (Kuo et al., 2023), such as symptom trajectories, treatment remission, and response.

## Supporting information

Zainal et al. supplementary material Zainal et al. supplementary material

## Data Availability

The authors will make the raw data underpinning this article’s conclusions accessible without undue restriction.

## Supplementary material

The supplementary material for this article can be found at http://doi.org/10.1017/S0033291725000340.
