# Supplementary material for: Capitalizing on natural language processing (NLP) to automate the evaluation of coach implementation fidelity in guided digital cognitive-behavioral therapy (GdCBT)
Source: Psychol Med. 2025 Apr 2;55:e106. doi: 10.1017/S0033291725000340 (PMC12094662; doi:10.1017/S0033291725000340)
Supplement: Zainal et al. supplementary material [file S0033291725000340sup001.docx]

# Online Supplementary Materials

# Capitalizing on Natural Language Processing (NLP) to Automate the Evaluation of Coach Implementation Fidelity in Guided Digital Cognitive-Behavioral Therapy (d-CBT)

## Machine learning methods

We rigorously assessed various machine learning algorithms to determine their suitability for predicting optimal (vs. non-optimal) coach behaviors, guided by theoretical considerations. As frequently mentioned in the literature (Domingos, 2000; Geman, Bienenstock, & Doursat, 1992; Hastie, Tibshirani, & Friedman, 2009), the bias-variance trade-off underscores the delicate balance in model selection. The ideal model should strike a balance between simplicity to avoid crude predictions and complexity to prevent overfitting, ensuring robust generalization to new, unseen data. In conjunction with other unique algorithmic characteristics, this principle served as a guiding force in our empirical investigation. As noted in the literature (Belkin, Hsu, Ma, & Mandal, 2019), enhancing a model's complexity and adaptability often enables it to capture finer-grained relationships but can lead to heightened susceptibility to data noise and overfitting (lack-of-generalizability issues).

**Gaussian Naïve Bayes.** The classification technique known as multinomial Naive Bayes operates by leveraging the principles of Bayes' theorem (Friedman, Linial, Nachman, & Pe'er, 2000). It ascribes a probability value to each message, quantifying its likelihood of membership within a particular category. This approach presupposes that the probability of one word's occurrence remains uncorrelated with the presence of another word within the confines of the same message.

**K-Nearest Neighbors (KNN).** The KNN algorithm was a contender in our evaluations, prized for its simplicity and the intuitive heuristic of classifying based on the proximity of observations to each other (Cover & Hart, 1967).

**Logistic regression.** Logistic regression was considered a candidate machine learning model primarily for its ability to provide interpretable log-odds coefficients for each feature, which is particularly advantageous in explanatory contexts. Additionally, its inherent compatibility with classification problems was a compelling factor in its consideration (Hastie et al., 2009).

**Multilayer perceptron (MLP).** The present study employed the multilayer perceptron (MLP) artificial neural network. The MLP is depicted as interconnected layers of nodes. The MLP comprises three layers: the input layer, the output layer, and one or more hidden layers. The application of an activation function to incoming weights on each node generates complex non-linear relationships between the network's inputs and outputs. In conjunction with the gradient descent optimization method, backpropagation is used to minimize a loss function. The network's weights are updated to minimize the loss function. After each iteration, the network weights underwent updates (Hush & Horne, 1993).

**Decision trees.** Decision trees (also called classification and regression trees; CART), a nonparametric approach, construct decision trees by considering predictors and their combinations that maximize the likelihood of distinguishing cases from non-cases. Decision trees can unveil interactions among extensive predictor sets without predefined specifications. Nevertheless, classification trees are more prone to overfitting than random forests (James, Witten, Hastie, & Tibshirani, 2013), as described below.

**Ada boost.** Ada boost, an abbreviation for adaptive boosting, is an ensemble machine learning approach that combines multiple models, often called "weak learners," to enhance overall performance and robustness (Freund & Schapire, 1997). This approach is achieved by assigning weights to both the weak learners and the data points. The algorithm compels weak learners to focus on challenging observations for correct classification. AdaBoost employs boosting, a sequential ensemble technique that assigns higher weights to misclassified cases, performs sampling without replacement, and mitigates the bias-variance tradeoff by aggregating weak or shallow learners through a voting process for prediction.

**Bagging.** The bagging ensemble predictive algorithm is utilized to amalgamate the predictive capabilities of multiple iterations of a base predictor, resulting in an aggregated predictor of superior accuracy (Kotsiantis, 2013). The multiple iterations of the base predictor are created through the bootstrap method, a widely utilized data resampling technique in statistical analysis (Breiman, 1996). The bagging ensemble predictive algorithm aims to diminish variance and prevent overfitting (González, García, Del Ser, Rokach, & Herrera, 2020). We utilized decision trees as the base predictor.

**Random forests.** The random forest algorithm extends the bagging method by combining bagging and feature randomness, forming an uncorrelated forest of decision trees (Breiman, 2001). Feature randomness, often referred to as feature bagging or "the random subspace method," produces a random feature subset, thereby ensuring minimal correlation among decision trees. This distinction stands as a pivotal divergence between decision trees and random forests. Whereas decision trees evaluate all potential feature splits, random forests opt for a subset of these features (Cutler, Cutler, & Stevens, 2012). It computes a locally optimized value for feature or data splitting using either the Gini coefficient or entropy value.

**Extra Trees.** Extra Trees, an abbreviation for "extremely randomized trees," is an ensemble technique in supervised machine learning that uses decision trees. Like the random forests algorithm, the Extra Trees algorithm generates numerous decision trees with random, non-repetitive sampling for each tree. This method produces a distinctive dataset for each tree, consisting of unique samples. For each tree, a random subset of features is chosen from the complete set of features. Unlike random forests, the defining and crucial element of Extra Trees is the randomized choice of a feature split value.

**Support vector machines (SVM).** SVMs construct a hyperplane within a high-dimensional space, intending to delineate clear boundaries between discrete classes (Pisner & Schnyer, 2020; Zhou et al., 2018). The principal aim is to ascertain a hyperplane that optimizes the separation distance from every class's nearest training data point stemming from their data transformation techniques, notably the kernel trick (Hastie et al., 2009; Hearst, Dumais, Osuna, Platt, & Scholkopf, 1998).

**Super Learner.** The Super Learner, an ensemble machine learning algorithm, combines all explored models and configurations for a predictive task to achieve predictions as effectively as, if not superior to, any individual model considered (van der Laan, Polley, & Hubbard, 2007). It exemplifies the broader technique known as "stacked generalization," commonly called "stacking" (Naimi & Balzer, 2018). The Super Learner algorithm begins by predefining k-fold data splits and subsequently assesses various algorithms and configurations using the same data split. The meta-model utilizes out-of-fold (out-of-sample) predictions as its input. Collectively, the out-of-fold predictions encapsulate a model's predictive prowess on unseen data, reflecting its capacity beyond the training set (van der Laan & Rose, 2011). Through training on out-of-sample predictions from other models, the meta-model simultaneously acquires the ability to refine individual out-of-sample predictions for each model and optimize the fusion of out-of-sample predictions from multiple models.

Table S1 *Coach best practice rubric implementation fidelity monitoring scale when delivering the d-CBT*

|  | **Yes/ No** |
| --- | --- |
| **A. Targets coach aims to meet at the BEGINNING** **and** **from time to time:** | |
| 1. Manages or clarifies user roles and expectations and review sessions |  |
| 1. Demonstrates interest in the user as a person |  |
| 1. Helps user clarify or specify primary concerns or goals |  |
| 1. Encourages use of SilverCloud |  |
| **B. Targets coach aims to meet for MOST review sessions (Compulsory):** | |
| 1. Reflects progress related to identified problem – anxiety, depression, ED |  |
| 1. Uses open-ended questions to guide treatment |  |
| 1. Reinforce positive steps (even if minuscule) |  |
| 1. Supports user autonomy |  |
| **C. Target(s) coach aims to meet for SOME review sessions (Optional):** | |
| 1. Connects to CBT formulation (thoughts-feelings-actions) |  |
| 1. Brainstorm solutions or alternatives |  |
| 1. Asks any other open-ended questions |  |
| 1. Answers any concerns users raise about SilverCloud modules |  |
| 1. Validate or normalizes user’s challenges |  |
| 1. Remind the user to put the things they are learning into practice (Self-efficacy promotion) |  |
| 1. Makes appropriate and personalized recommendation content and tools used - giving a clear rationale for why (Set homework) |  |
| 1. Give clear deadlines for the completion of tasks - conscious of tone and avoiding being too directive (Set homework) |  |
| **D. Things that coach should NOT do:** |  |
| 1. Reinforces bad behavior or mindsets |  |
| 1. Over-shares personal information |  |
| 1. Interprets underlying motives |  |
| 1. Gives direct and specific advice in patronizing, condescending, or other unhelpful ways |  |

*Note.* d-CBT, digital cognitive-behavioral therapy; ED, eating disorders.

Table S2 *Coach-to-user example messages to make ratings for each fidelity behavior code*

| **Code** | **Example** |
| --- | --- |
| **A1**: Manages or clarifies user roles and expectations | *“… Before I get to that, I just want to make sure you remember a few key details about how this will work. The goal of this program is to help you learn how to apply the skills taught in each module to your daily life. …”* |
| **A2**: Demonstrates interest in the user as a person | *“Hi [name]! How are you doing? I hope all is well!”* |
| **A3**: Helps user clarify or specify primary concerns or goals | *“…One of your goals is to utilize daily practice. This is great! What do you think would be helpful to practice daily? …”* |
| **A4**: Encourages use of SilverCloud | “…*You have been making really great progress and I am excited to see what you will be able to do in the next 4 weeks!* …” |
| **B1:** Reflects progress related to identified problem | “…*I noticed that your questionnaire for anxiety improved considerably. How do you feel about your progress?*...” |
| **B2**: Uses open-ended questions to guide treatment | “…*What do you think would be helpful to practice daily?*” |
| **B3:** Reinforce positive steps (even if minuscule) | “…*Great work over the past few days. You used 4 tools, logged in 4 times, and completed the Understanding Depression unit*. …” |
| **B4**: Supports user autonomy | “… *Think about which tool or tools you want to use daily to move forward with your goal*. …” |
| **B5**: Connects to CBT formulation (thoughts-feelings-actions) | “… *The ‘Understanding Feelings’ module is available for you...to understand the role that your emotions play in the Thoughts, Feelings, Behaviors (TFB) cycle*. …” |
| **B6**: Brainstorm solutions or alternatives | “… *When do you think CBT may have been and will be helpful to you?* …” |
| **B7**: Asks any other open-ended questions | “… *Do you have any questions on this unit?* …” |
| **B8**: Answers any concerns users raise about SilverCloud modules | “… *Please know that I only see your activity on your set review date, so if you need any immediate support, you may want to contact the crisis text line or your school’s counselling center*. …” |
| **C1**: Validate or normalizes user’s challenges | “…*I understand that spotting your own thoughts can be difficult at first, but with practice it will soon become easier*. …” |
| **C2**: Remind the user to put the things they are learning into practice | “…*You mentioned that you’ve started practicing your meditation exercises in your daily life and are already noticing the benefits, which is fantastic!*...” |
| **C3**: Makes appropriate and personalized recommendation content and tools used | “…*This week you revisited the Safety Behaviours tool...You can then use the Hierarchy of Fears Tool to plan small, manageable steps to change these avoidant behaviours*. …” |
| **C4**: Give clear deadlines for the completion of tasks | “…*I’ll check back in with you on Wednesday, June 2nd*. …” |
| **D1**: Reinforces bad behavior or mindsets | *“Don’t worry about the missed logins, just do what you can when you have time*.” |
| **D2**: Over-shares personal information | “*No problem at all if you’ve been busy! I know I also struggled with consistency when I started this kind of work, so don’t worry too much about it*. …” |
| **D3**: Interprets underlying motives | “…*Thanks for the message - no problem if you didn’t check in! It seems like you might be feeling overwhelmed lately, so just reach out when it feels right*. …” |
| **D4**: Gives direct and specific advice in unhelpful ways | “…*Thanks for your message and don’t worry too much about not logging in! Try to log in whenever you feel like it*. …” |

*Note.* This table expands on Table S1.

Table S3 *Correlations of emotion word frequency count among three sentiment lexicons*

|  | 1 | 2 | 3 | 4 | 5 | 6 |
| --- | --- | --- | --- | --- | --- | --- |
| 1. Negative (Bing) | – |  |  |  |  |  |
| 1. Positive (Bing) | .975^***^ | – |  |  |  |  |
| 1. Negative (NFC) | .994^***^ | .969^***^ | – |  |  |  |
| 1. Positive (NFC) | .974^***^ | .983^***^ | .964^***^ | – |  |  |
| 1. Negative (AFINN) | .971^***^ | .943^***^ | .972^***^ | .953^***^ | – |  |
| 1. Positive (AFINN) | .973^***^ | .957^***^ | .972^***^ | .961^***^ | .989^***^ | – |

*Note.* ^***^ *p* < .001.

Table S4 *Summary of codes in the Motivational Interviewing Skill Code (MISC) to draw comparisons*

| 1. ADP: Advise with permission |
| --- |
| 1. ADW: Advise without permission |
| 1. AF: Affirm |
| 1. CO: Confront |
| 1. DI: Direct |
| 1. EC: Emphasize Control |
| 1. FA: Facilitate |
| 1. FI: Filler |
| 1. GI: Giving Information |
| 1. QUC: Question - Closed |
| 1. QUO: Question - Open |
| 1. RCP: Raise Concern with permission |
| 1. RCP: Raise Concern without permission |
| 1. RES: Reflect - Simple |
| 1. REC: Reflect - Complex |
| 1. RF: Reframe |
| 1. SU: Support |
| 1. ST: Structure |
| 1. WA: Warn |

*Note.* Details of this fidelity coding manual can be accessed from Miller, Moyers, Ernst, and Amrhein (2008).

Table S5 *Summary of codes in the Motivational Interviewing Treatment Integrity (MITI) to draw comparisons*

| 1. Giving Information (GI) |
| --- |
| 1. Persuade (P) |
| 1. Persuade with Permission (PW) |
| 1. Question (Q) |
| 1. Simple Reflection (SR) |
| 1. Complex Reflection (CR) |
| 1. Affirm (AF) |
| 1. Seeking Collaboration (Seek) |
| 1. Emphasizing Autonomy (Emphasize) |
| 1. Confront (Confront) |

*Note.* Details of this fidelity coding manual can be accessed from Moyers, Manuel, and Ernst (2015).

Table S6 *Summary of codes in the Cognitive Therapy Scale (CTS) and Cognitive Therapy Scale-Revised (CTS-R)*

| 1. Agenda setting and adherence 2. Feedback 3. Collaboration 4. Pacing and efficient use of time 5. Interpersonal effectiveness 6. Charisma/flair 7. Eliciting appropriate emotional expression 8. Eliciting key cognitions 9. Eliciting behaviors 10. Guided discovery 11. Conceptual integration 12. Application of change methods 13. Homework setting 14. Non-verbal behavior |
| --- |

*Note.* Details of this fidelity coding manual can be accessed from Blackburn et al. (2001).

Table S7 *Summary of codes in the Competence and Adherence Scale for Cognitive Behavioral Therapy (CAS-CBT)*

| 1. **Homework Activity Review and Presenting New Homework Tasks (adherence)**: Scored based on the time used by the therapist to review previous homework and plan new homework. 2. **Structure and Progress (adherence)**: Scored based on whether the therapist presents and follows an agenda and uses time efficiently to meet session goals. 3. **Parental Involvement (adherence)**: Scored only if parental involvement is part of the treatment protocol, evaluating the extent of parental participation as per protocol. 4. **Cognitive Behavior Therapy Structure (competence for items 1–3)**: Scored based on skill level in CBT structure, with a score of 6 (excellent) requiring clear, engaging presentation of homework, efficient time use, structured session content, and well-communicated goals to both youth and parents (if applicable). 5. **Positive Reinforcement (adherence)**: Scored based on the degree to which the therapist uses positive reinforcement (e.g., praise, nodding, rewards) for youth’s behavior. 6. **Collaboration (adherence)**: Scored based on the therapist’s facilitation of a collaborative environment where responsibility is shared appropriately with the youth. 7. **Flexibility (competence)**: Scored on the therapist's ability to tailor the intervention to the youth’s condition, mood, level of engagement, or developmental stage. 8. **Process and Relational Skills (competence for items 5 and 6)**: Scored based on skill in using encouragement and praise effectively to foster desired behaviors and motivate active engagement; requires respectful feedback, collaboration, and warmth. 9. **Session Goal 1 (adherence)**: Scored based on the extent to which a specific goal for the session is achieved. 10. **Session Goal 2 (adherence)**: Scored based on the extent to which another specific goal for the session is achieved. 11. **Session Goals (competence for items 9 and 10)**: Scored based on the skill level in presenting session goals, with a score of 6 (excellent) requiring the therapist to engage the youth effectively and use relevant examples linked to the youth’s specific issues. |
| --- |

*Note.* Details of this fidelity coding manual can be accessed from Bjaastad et al. (2016).

Table S8 *Summary of codes in the ACE Treatment Integrity Measure (ATIM)*

| For each item below, circle 0 if absent or 1 if present for this therapy session: |
| --- |
| **Specific Therapy Techniques:** |
| 1. 0 1 Therapist develops a formulation with the client (e.g., makes links between past events, current situation, and symptoms or relates past events, current situation, and symptoms to a cognitive model). 2. 0 1 Assessment/information gathering concerning symptoms, past life events. 3. 0 1 Therapist engages with client in setting goals for therapy. 4. 0 1 Therapist gives client psychoeducation. 5. 0 1 Therapist redirects from unresolved conflicts to a neutral topic. 6. 0 1 Therapist uses motivational interviewing techniques. 7. 0 1 Therapist sets or follows-up homework. 8. 0 1 Therapist redirects from discussion about symptoms to a neutral topic. 9. 0 1 Therapist focuses on key cognitions. 10. 0 1 Therapist and client work on thought records. 11. 0 1 Therapist and client engage in role reversal exercises. 12. 0 1 Therapist chooses the most neutral line of questioning. 13. 0 1 Therapist guides the client in perspective taking. 14. 0 1 Therapist engages in cognitive challenging. 15. 0 1 Therapist reacts minimally to loaded speech (e.g., symptoms, conflicts). 16. 0 1 Therapist and client work on cognitive coping strategies for specific symptoms. 17. 0 1 Therapist redirects from identity issues to a neutral topic. 18. 0 1 Therapist uses guided discovery/Socratic questioning. 19. 0 1 Therapist sets or discusses behavioral or cognitive experiments. 20. 0 1 Therapist engages in cognitive restructuring. 21. 0 1 Therapist guides client in identifying triggers. 22. 0 1 Therapist gives client education about the therapy process. 23. 0 1 Therapist guides client in problem-solving. 24. 0 1 Therapist engages the client in exposure training. 25. 0 1 Therapist engages the client in relaxation training. 26. 0 1 Therapist and client work on behavioral coping strategies for specific symptoms. |
| **General Therapy Techniques:** |
| 1. 0 1 Collaboration. 2. 0 1 Empathy. 3. 0 1 Professionalism. 4. 0 1 Therapist direction of the therapeutic process. |

*Note.* CBT, cognitive-behavioral therapy. Details of this fidelity coding manual can be accessed from Bendall et al. (2015).

Table S9 *Summary of codes in the Cognitive Processing Therapy (CPT) - Therapist Adherence and Competence Protocol*

| **Specific Session Elements (Rate for Adherence and Competence)** |
| --- |
| 1. Educated client on PTSD and the 4 symptom clusters. |
| 1. Educated client about the fight-flight response with examples. |
| 1. Educated client on cognitive theory (e.g., “just world” myth). |
| 1. Provided education on types of emotions. |
| 1. Provided treatment rationale for recognizing/modifying thoughts. |
| 1. Explained stuck points, introduced Stuck Point Handout. |
| 1. Assigned Impact Statement as practice assignment. |
| **Essential but Not Unique Elements (Competence Ratings)** |
| 1. Established rapport. |
| 1. Reviewed homework and discussed barriers. |
| 1. Structured the session and used time effectively. |
| **Proscribed Elements (Yes/No Ratings)** |
| 1. Significant problems arose leading to departure from the agenda. |
| 1. Therapist implemented interventions not included in the manual. |
| 1. Therapist engaged in more than 15 minutes of off-task discussion. |

*Note.* PTSD, post-traumatic stress disorder. Details of this fidelity coding manual can be accessed from Marques et al. (2019) and Nishith and Resick (1994).

Table S10 *Summary of code in the Cognitive Therapy Adherence and Competence Scale*

| **Cognitive therapy structure** |
| --- |
| 1. **Agenda:** identified important target problems; prioritized and followed the agenda. 2. **Mood Check**: asked about mood; followed up with clarification; added important mood-related concerns to the agenda and addressed them. 3. **Bridge from Previous Visit:** discussed previous session with the patient; emphasized key issues; related it to current agenda items; added unresolved issues to the current agenda. 4. **Inquired About Ongoing Problem:** inquired about ongoing difficulties (e.g., urges, cravings, drug use) and followed up with appropriate responses and interventions. 5. **Reviewing Previous Homework:** reviewed previous homework or discussed incomplete homework. 6. **Assigning New Homework:** collaboratively assigned new homework; discussed, planned, and practiced it within the session. 7. **Capsule Summaries:** provided meaningful capsule summaries; checked for accuracy and revised when appropriate. 8. **Patient Summary and Feedback:** asked for summary and feedback throughout the session; responded positively and supportively; adjusted behavior based on patient’s feedback. 9. **Focus/Structure:** used time effectively by directing conversation flow and redirecting as needed; ensured session was well-paced, focused, and structured. |
| **Development of a collaborative therapeutic relationship** |
| 1. **Socialization to Cognitive Therapy:** described relevant model, concepts, process, and structure; applied these timely; checked understanding and elicited feedback. 2. **Warmth/Genuineness/Congruence:** appeared optimally warm, genuine, caring, and congruent. 3. **Acceptance/Respect:** displayed full acceptance, respect, and non-judgmental attitude. 4. **Attentiveness:** was attentive to important and subtle cues. 5. **Accurate Empathy:** demonstrated empathy skills and insight; shared appropriately with the patient. 6. **Collaboration:** shared responsibility for defining problems and potential solutions; functioned as a team with the patient. |
| **Development and application of the case conceptualization** |
| 1. **Eliciting Automatic Thoughts:** elicited automatic thoughts (ATs) and related them to the patient’s problems. 2. **Eliciting Core Beliefs and Schemas:** elicited core beliefs and schemas; effectively linked these to the patient’s issues. 3. **Eliciting Meaning/Understanding/Attributions:** asked for meaning of significant events and beliefs; followed up as needed. 4. **Addressing Key Issues:** raised important key issues and related them to schemas, core beliefs, conditional beliefs, automatic thoughts, emotions, and behaviors. 5. **Case Conceptualization:** Linking Past to Present: inquired about developmental processes when appropriate; accurately linked these to current beliefs, thoughts, emotions, and behaviors; elicited feedback on accuracy and usefulness. 6. **Sharing the Conceptualization:** provided the patient with a conceptualization of problems; elicited feedback on accuracy and usefulness. |
| **Cognitive and behavioral techniques** |
| 1. **Guided Discovery:** used open-ended questions, reflective, confrontive, and interpretive responses to guide patient understanding of important issues. 2. **Asking for Evidence/Alternative Views:** asked the patient for evidence of maladaptive beliefs; where appropriate, explored alternative views and followed up. 3. **Use of Alternative Cognitive and Behavioral Techniques:** effectively selected and applied standardized cognitive and behavioral methods (specify technique as needed). 4. **Overall Performance as a Cognitive Therapist:** demonstrated excellent performance; practiced cognitive therapy at a highly proficient level; exhibited in-depth knowledge of the relevant treatment manual, applying the cognitive case formulation with ease and flexibility; represented state-of-the-art cognitive therapy. |

*Note.* Details of this fidelity coding manual can be accessed from Barber, Liese, and Beck (1995)

References

Barber, J. P., Liese, S. S., & Beck, A. T. (1995). *Cognitive Therapy Adherence and Competence Scale (CTACS)*: University of Kansas.

Belkin, M., Hsu, D., Ma, S., & Mandal, S. (2019). Reconciling modern machine-learning practice and the classical bias-variance trade-off. *Proceedings of the National Academy of Sciences of the United States of America, 116*, 15849-15854. doi:10.1073/pnas.1903070116

Bendall, S., Allott, K., Jovev, M., Marois, M. J., Killackey, E. J., Gleeson, J. F., . . . Jackson, H. J. (2015). Therapy contamination as a measure of therapist treatment adherence in a trial of cognitive behaviour therapy versus befriending for psychosis. *Behavioural and Cognitive Psychotherapy, 43*, 314-327. doi:10.1017/s1352465813000921

Bjaastad, J. F., Haugland, B. S., Fjermestad, K. W., Torsheim, T., Havik, O. E., Heiervang, E. R., & Ost, L. G. (2016). Competence and Adherence Scale for Cognitive Behavioral Therapy (CAS-CBT) for anxiety disorders in youth: Psychometric properties. *Psychological Assessment, 28*, 908-916. doi:10.1037/pas0000230

Blackburn, I.-M., James, I. A., Milne, D. L., Baker, C., Standart, S., Garland, A., & Reichelt, F. K. (2001). The Revised Cognitive Therapy Scale (CTS-R): Psychometric properties. *Behavioural and Cognitive Psychotherapy, 29*, 431-446. doi:10.1017/s1352465801004040

Breiman, L. (2001). Random forests. *Machine Learning, 45*, 5-32. doi:10.1023/A:1010933404324

Breiman, L. (1996). Bagging predictors. *Machine Learning, 24*, 123-140. doi:10.1007/BF00058655

Cover, T., & Hart, P. (1967). Nearest neighbor pattern classification. *IEEE Transactions on Information Theory, 13*, 21-27. doi:10.1109/TIT.1967.1053964

Cutler, A., Cutler, D. R., & Stevens, J. R. (2012). Random forests. In C. Zhang & Y. Ma (Eds.), *Ensemble machine learning: Methods and applications* (pp. 157-175). Boston, MA: Springer US. doi: 10.1007/978-1-4419-9326-7_5

Domingos, P. M. (2000). *A unified bias-variance decomposition for zero-one and squared loss.* Paper presented at the AAAI/IAAI.

Freund, Y., & Schapire, R. E. (1997). A decision-theoretic generalization of on-line learning and an application to boosting. *Journal of Computer and System Sciences, 55*, 119-139. doi:10.1006/jcss.1997.1504

Friedman, N., Linial, M., Nachman, I., & Pe'er, D. (2000). Using Bayesian networks to analyze expression data. *Journal of Computational Biology, 7*, 601-620. doi:10.1089/106652700750050961

Geman, S., Bienenstock, E., & Doursat, R. (1992). Neural networks and the bias/variance dilemma. *Neural Computation, 4*, 1-58. doi:10.1162/neco.1992.4.1.1

González, S., García, S., Del Ser, J., Rokach, L., & Herrera, F. (2020). A practical tutorial on bagging and boosting based ensembles for machine learning: Algorithms, software tools, performance study, practical perspectives and opportunities. *Information Fusion, 64*, 205-237. doi:10.1016/j.inffus.2020.07.007

Hastie, T., Tibshirani, R., & Friedman, J. (2009). *The elements of statistical learning*. New York, NY: Springer Science & Business Media.

Hearst, M. A., Dumais, S. T., Osuna, E., Platt, J., & Scholkopf, B. (1998). Support vector machines. *IEEE Intelligent Systems, 13*, 18-28. doi:10.1109/5254.708428

Hush, D. R., & Horne, B. G. (1993). Progress in supervised neural networks. *IEEE Signal Processing Magazine, 10*, 8-39. doi:10.1109/79.180705

James, G., Witten, D., Hastie, T., & Tibshirani, R. (2013). *An introduction to statistical learning* (Vol. 103). New York, NY: Springer New York.

Kotsiantis, S. B. (2013). Bagging and boosting variants for handling classifications problems: A survey. *Knowledge Engineering Review, 29*, 78-100. doi:10.1017/s0269888913000313

Marques, L., Valentine, S. E., Kaysen, D., Mackintosh, M. A., Dixon De Silva, L. E., Ahles, E. M., . . . Wiltsey-Stirman, S. (2019). Provider fidelity and modifications to cognitive processing therapy in a diverse community health clinic: Associations with clinical change. *Journal of Consulting and Clinical Psychology, 87*, 357-369. doi:10.1037/ccp0000384

Miller, W. R., Moyers, T. B., Ernst, D., & Amrhein, P. (2008). *Manual for the Motivational Interviewing Skill Code (MISC): Version 2.1*. Unpublished manual.

Moyers, T. B., Manuel, J. K., & Ernst, D. (2015). *Motivational Interviewing Treatment Integrity Coding Manual 4.2.1*. Unpublished manual.

Naimi, A. I., & Balzer, L. B. (2018). Stacked generalization: an introduction to super learning. *European Journal of Epidemiology, 33*, 459-464. doi:10.1007/s10654-018-0390-z

Nishith, P., & Resick, P. A. (1994). *Cognitive processing therapy (CPT): Therapist adherence and competence protocol*. Unpublished instrument. University of Missouri–St. Louis, St. Louis, MO.

Pisner, D. A., & Schnyer, D. M. (2020). Support vector machine *Machine Learning* (pp. 101-121). doi: 10.1016/b978-0-12-815739-8.00006-7

van der Laan, M. J., & Rose, S. (2011). *Targeted learning: causal inference for observational and experimental data*. New York, NY: Springer.

van der Laan, M. J., Polley, E. C., & Hubbard, A. E. (2007). Super learner. *Statistical Applications in Genetics and Molecular Biology, 6*, Article25. doi:10.2202/1544-6115.1309

Zhou, C., Cheng, Y., Ping, L., Xu, J., Shen, Z., Jiang, L., . . . Xu, X. (2018). Support vector machine classification of obsessive-compulsive disorder based on whole-brain volumetry and diffusion tensor imaging. *Frontiers in Psychiatry, 9*, 524. doi:10.3389/fpsyt.2018.00524
